# Many locks to one key: *N*-acetyl­neuraminic acid binding to proteins

**DOI:** 10.1107/S2052252524005360

**Published:** 2024-07-04

**Authors:** KanagaVijayan Dhanabalan, YiYang Cheng, Trung Thach, Ramaswamy Subramanian

**Affiliations:** a Department of Biological Sciences Purdue University West Lafayette IN47907 USA; b Weldon School of Biomedical Engineering Purdue University West Lafayette IN47907 USA; Chinese Academy of Sciences, China

**Keywords:** sialic acids, *N*-acetyl neuraminic acid, Neu5Ac, Gram-negative bacteria, binding sites, drug discovery, protein structures, molecular recognition

## Abstract

Sialic acids play crucial roles in cell surface glycans of both eukaryotic and prokaryotic organisms, mediating various biological processes, including cell–cell interactions, development, immune response, oncogenesis and host–pathogen interactions. This review focuses on the β-anomeric form of *N*-acetyl­neuraminic acid (Neu5Ac), particularly its binding affinity towards various proteins, as elucidated by solved protein structures. Specifically, we delve into the binding mechanisms of Neu5Ac to proteins involved in sequestering and transporting Neu5Ac in Gram-negative bacteria, with implications for drug design targeting these proteins as antimicrobial agents. Unlike the initial assumptions, structural analyses revealed significant variability in the Neu5Ac binding pockets among proteins, indicating diverse evolutionary origins and binding modes. By comparing these findings with existing structures from other systems, we can effectively highlight the intricate relationship between protein structure and Neu5Ac recognition, emphasizing the need for tailored drug design strategies to inhibit Neu5Ac-binding proteins across bacterial species.

## Abstract 2

In structural biology, the analogy of a key (ligand) fitting a lock (protein) is commonly used to describe the binding process. In this context, we illustrate the evolutionary development of diverse locks that exhibit specific binding to a shared key: Neu5Ac. The intricate specificity of the interaction between various locks and the common key (Neu5Ac) is explored in our review.

## 1. Sialic acids

**Figure 1:**
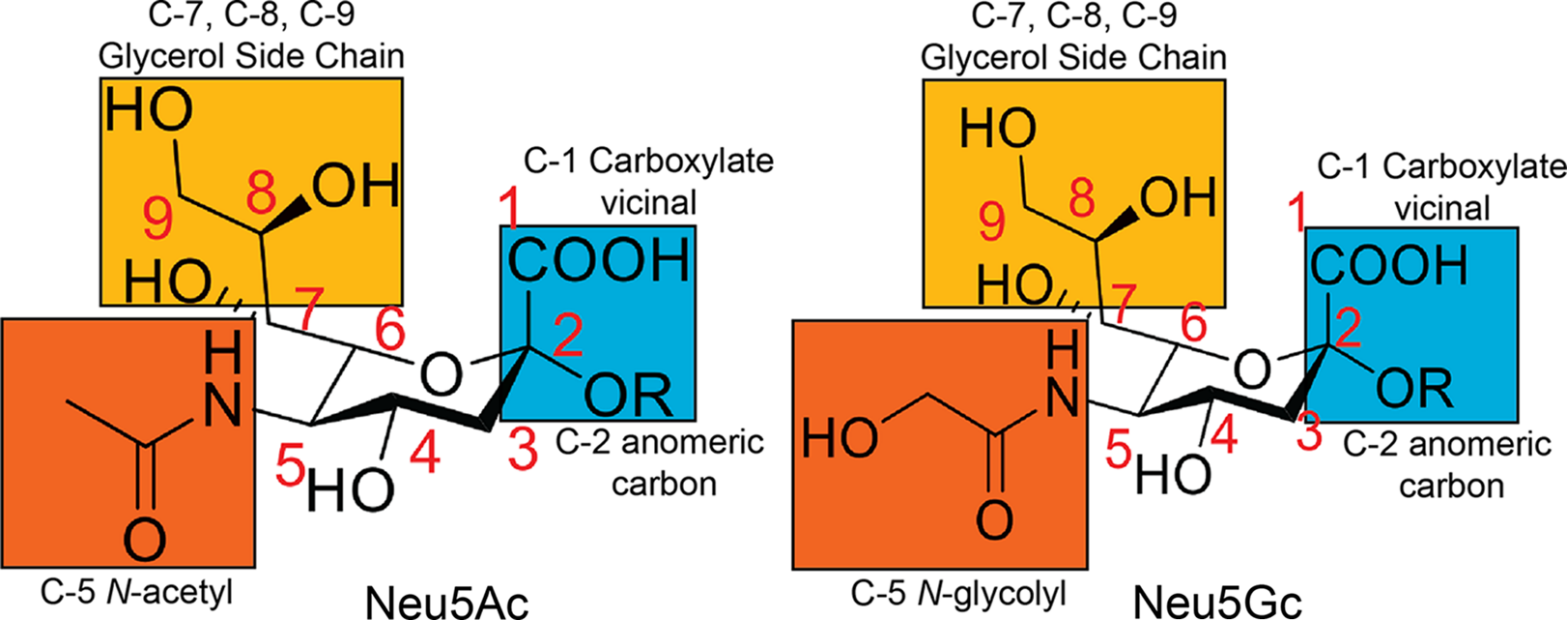
Structures of the two most common forms of sialic acid: Neu5Ac and Neu5Gc.

Sialic acids are a group of over 50 different types of nine-carbon sugars (Varki, 1992 ▸). One of the most widely studied sialic acids is 2-keto-3-de­oxy-5-acetamido d-*glycero*-d-*galacto*-nonulosonic acid, also known as *N*-acetyl neuraminic acid (Neu5Ac). The diversity of sialic acids is brought about by the generation of amine- or hydroxyl-linked modifications. The most common modification is the O-acetyl­ation of carbons 4, 7, 8 and 9 (Angata & Varki, 2002 ▸). The other major form is *N*-glycolyl neuraminic acid (Neu5Gc), which is generated by hy­droxy­lation of the 5-acetamido position in Neu5Ac (Fig. 1 ▸). Neu5Gc is absent in humans due to a frameshift mutation in the cytidine-5′-monophospho-*N*-acetyl­neuraminic acid (CMP-Neu5Ac) hy­droxy­lase gene, but is present in our closest primate relatives (Angata *et al.*, 2001 ▸).

Sialic acids are typically found at the terminal position in both eukaryotic and prokaryotic cell surface glycans. They are (α2–3)- or (α2–6)-glycosidically linked to galactosides or (α2–6)-linked to *N*-acetyl­galactosamine (Boons & Demchenko, 2000 ▸). Polysialic acids of Neu5Ac and Neu5Gc, characterized by (α2–8), (α2–9) or alternating (α2–8)/(α2–9) glycosidic linkages, have been found in the glycoproteins of neural cell adhesion molecules (NCAMs) in fish eggs and in the capsules of certain bacteria, such as *Neisseria meningitidis* group B (Sato & Kitajima, 2013 ▸).

In humans, sialic acid (Neu5Ac) is the most abundant sugar in the central nervous system, hence the name neuraminic acid (Haines-Menges *et al.*, 2015 ▸). In the brain, polysialic acids coat neurons, glia and ganglia. Another major reservoir of sialic acids is the gastrointestinal tract, followed by other mucin-rich regions such as the lungs and vagina (Schnaar *et al.*, 2014 ▸). Neu5Ac often occurs at the terminal positions of glycoconjugates; is involved in cell–cell interactions; and mediates processes such as development, immune response, oncogenesis and host–pathogen interactions (Varki, 2017 ▸; Jennings *et al.*, 2022 ▸).

Notably, both the α-anomer and β-anomer forms of Neu5Ac exist in biological systems. In this review, we focus on the β-anomer form of Neu5Ac to identify the similarities and the significance of the protein environment to the binding affinity towards the β-anomer of Neu5Ac in solved protein structures. Over a decade ago, the laboratory started working on the sequestering, transport and catabolism of Neu5Ac in bacteria that persist in different niches of the human body. Many of these proteins bind to Neu5Ac, and analysing the nature of these binding pockets will provide crucial insights for designing drugs to inhibit these proteins, often cited as possible new antimicrobial targets. Our naïve hypothesis was that, given that the sequestering proteins, transporters and the first enzymes that utilize Neu5Ac all bind Neu5Ac, we should be able to design inhibitors that will bind to all these proteins. As the structures began to be determined, it was apparent that each of these proteins probably evolved from a different protein and bound to Neu5Ac differently. Here, we review how Neu5Ac binds to different proteins in this class of Gram-negative bacteria and compare them with other published structures of β-Neu5Ac binding in other systems. The collective data reveal substantial diversity among the locks binding to the identical key (Neu5Ac).

## 2. Bacterial sialometabolism

The first evidence that bacteria possess sialic acids was obtained from *E. coli* isolates in 1958 (Barry, 1959 ▸). Sialidases are enzymes that cleave sialic acids from their glycoconjugates in bacteria, suggesting that they utilize sialic acids. In bacteria, sialometabolism involves the following: (1) biosynthesis and scavenging of sialic acids for sialylation of their cell surface; and (2) sialic acid catabolism, utilizing it as a carbon and nitro­gen source.

### 2.1. Utilization of sialic acid by bacteria to evade host immune response

Studies have shown that *H. influenzae*, *E. coli*, *C. jejuni*, *P. multocida*, *H. ducreyi*, *N. meningitidis* and *N. gonorrhoeae* can coat the capsular polysaccharide or lipooligosaccharide layer with Neu5Ac to evade the host immune system via molecular mimicry (Vimr *et al.*, 2000 ▸). To do so, they evolved at least four mechanisms to acquire sialic acids, as outlined below.

#### 2.1.1. *De novo* synthesis

*E. coli* and *N. meningitidis* utilize this mechanism to obtain sialic acids for cell surface sialylation. Neu5Ac is synthesized from uridine diphosphate, *N*-acetyl glucosamine (UDP–GlcNAc) by hydrolyzing UDP–GlcNAc-2. The ManNAc produced is condensed with phospho­enol pyruvate to yield Neu5Ac (Vimr & Lichtensteiger, 2002 ▸). Neu5Ac is then activated to form CMP–Neu5Ac through the action of CMP–Neu5Ac synthetase. Specific sialyltransferases add sialic acids to the capsular polysaccharide or lipooligosaccharide layers (Vimr *et al.*, 2004 ▸).

#### 2.1.2. Precursor scavenging

Neu5Ac is scavenged from the environment through this mechanism. This requires sialic acid transporters to transport sialic acid from the environment to the cytoplasm. Once the scavenged sialic acid is transported into the cell, CMP–Neu5Ac is synthesized and transferred onto the LPS/LOS by sialyltransferases (Vimr & Lichtensteiger, 2002 ▸; Apicella, 2012 ▸). *H. influenza* is an example of a pathogen that utilizes this mechanism. This review primarily focuses on the pathways for Neu5Ac sequestration, import and utilization by Gram-negative bacteria.

#### 2.1.3. Donor scavenging

To date, this mechanism of directly scavenging host CMP–Neu5Ac has been reported only in *N. gonorrhoea*, as it lacks the machinery for the biosynthesis of sialic acids and CMP–Neu5Ac production (Vimr & Lichtensteiger, 2002 ▸).

#### 2.1.4. Trans-sialidase activity

The protozoan *T. cruzi* secretes a surface α2–3 *trans*-sialidase that cleaves and transfers host α2–3 sialyl conjugates directly to the terminal galactose of its mucins (Vimr & Lichtensteiger, 2002 ▸).

### 2.2. Utilization of sialic acid as a carbon and nitro­gen source

The other utilization of sialic acids is as a source of carbon and nitro­gen. Bacteria colonizing mucin-rich environments have a low supply of glucose, especially in the gastrointestinal tract (Jeong *et al.*, 2009 ▸). Bacteria that have evolved the sialic acid catabolism pathway have considerable advantages in colonizing sialic acid-rich environments (Jeong *et al.*, 2009 ▸; Almagro-Moreno & Boyd, 2010 ▸; Olson *et al.*, 2013 ▸). Sialic acid is degraded to fructose-6-phosphate, which subsequently undergoes glycolysis. The intermediate GlcNAc-6-P can also be a precursor of cell wall biosynthetic pathways. The enzymes involved in sialic acid catabolism are generally found in the tightly regulated nan–nag operon (Almagro-Moreno & Boyd, 2010 ▸).

## 3. Transport of sialic acids

**Figure 2:**
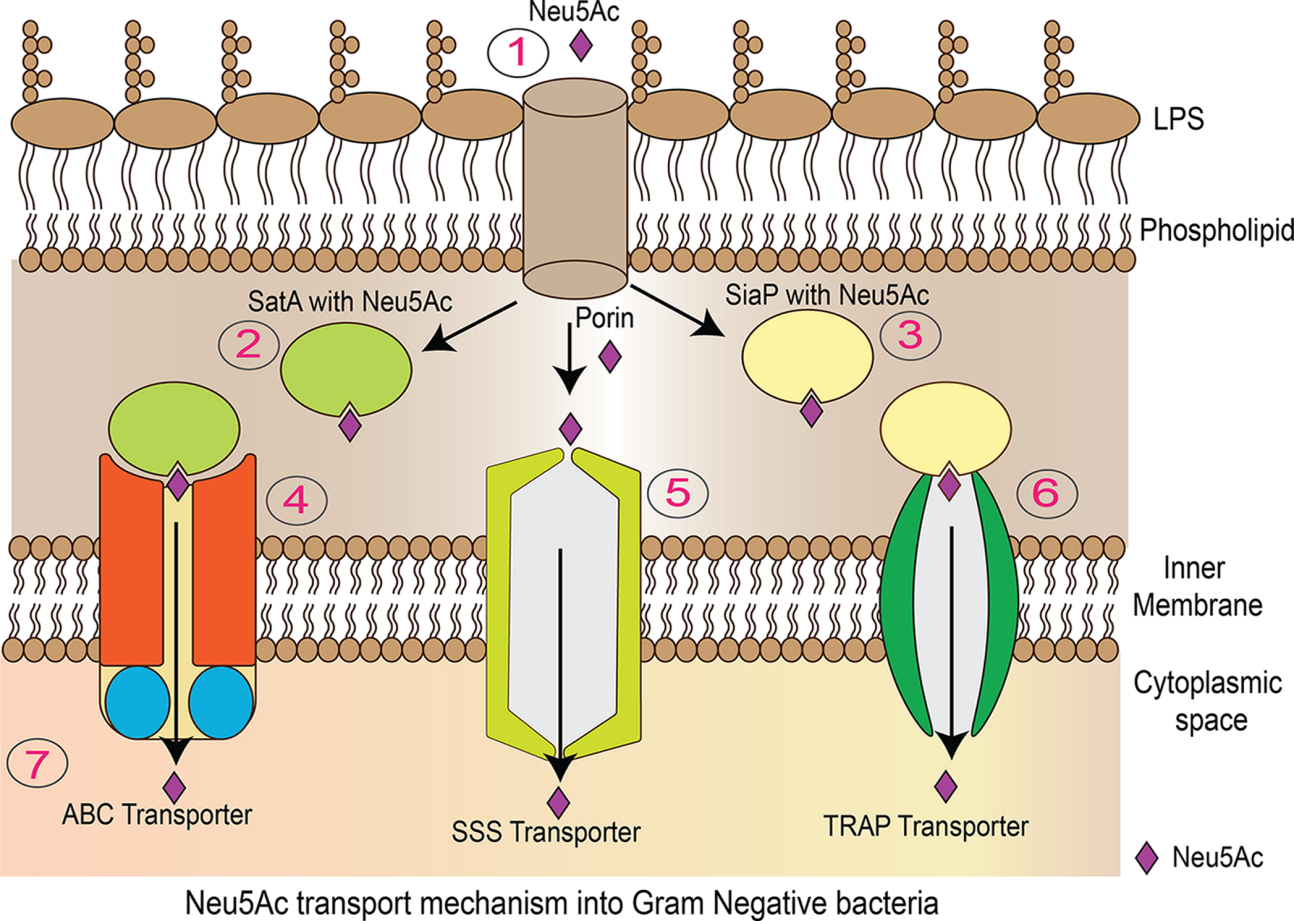
Neu5Ac transport mechanism in Gram-negative bacteria. (1) Porins located in the outer membrane facilitate the transport of Neu5Ac into the periplasmic space. (2) and (4) SatA proteins bind to Neu5Ac molecules and are then associated with the ABC transporter. SiaP proteins (3) also bind to Neu5Ac and assist TRAP transporters (6) in translocating Neu5Ac into the cytoplasm. (5) The SSS transporter facilitates the movement of cargo molecules across the membrane using the energy provided by an ion gradient.

For bacteria to utilize sialic acids obtained from host scavenging, sialic acid must be transported across the cell membrane (Fig. 2 ▸). Transport from the outer membrane to the periplasmic space occurs through porins (NanC). Transport into bacterial cells from the periplasmic space occurs via two types of transporters: primary active transporters [ATP synthase binding cassette transporters (ABC transporters)] and secondary active transporters. Three distinct secondary active transporters of Neu5Ac have been identified: (1) a single-component sodium solute symporter (SSS), (2) a tripartite ATP-independent periplasmic transporter (TRAP) and (3) a single-component sugar proton symporter (NanT) (Thomas, 2016 ▸).

### 3.1. Primary active transporters: ABC transporters

ABC transporters, known as primary active transporters, use energy driven by ATP hydrolysis to import various substrates. In bacteria, ABC transporters are generally assembled into four subunits: two transmembrane domains (TMDs) and two nucleotide-binding domains (NBDs). In Gram-negative bacteria, importers contain a substrate-binding protein in the periplasm that traps the substrate and delivers it to the TMD. These proteins transport a diverse range of substrates and thus lack sequence similarity in their TMDs (Dawson & Locher, 2006 ▸).

**Figure 3:**
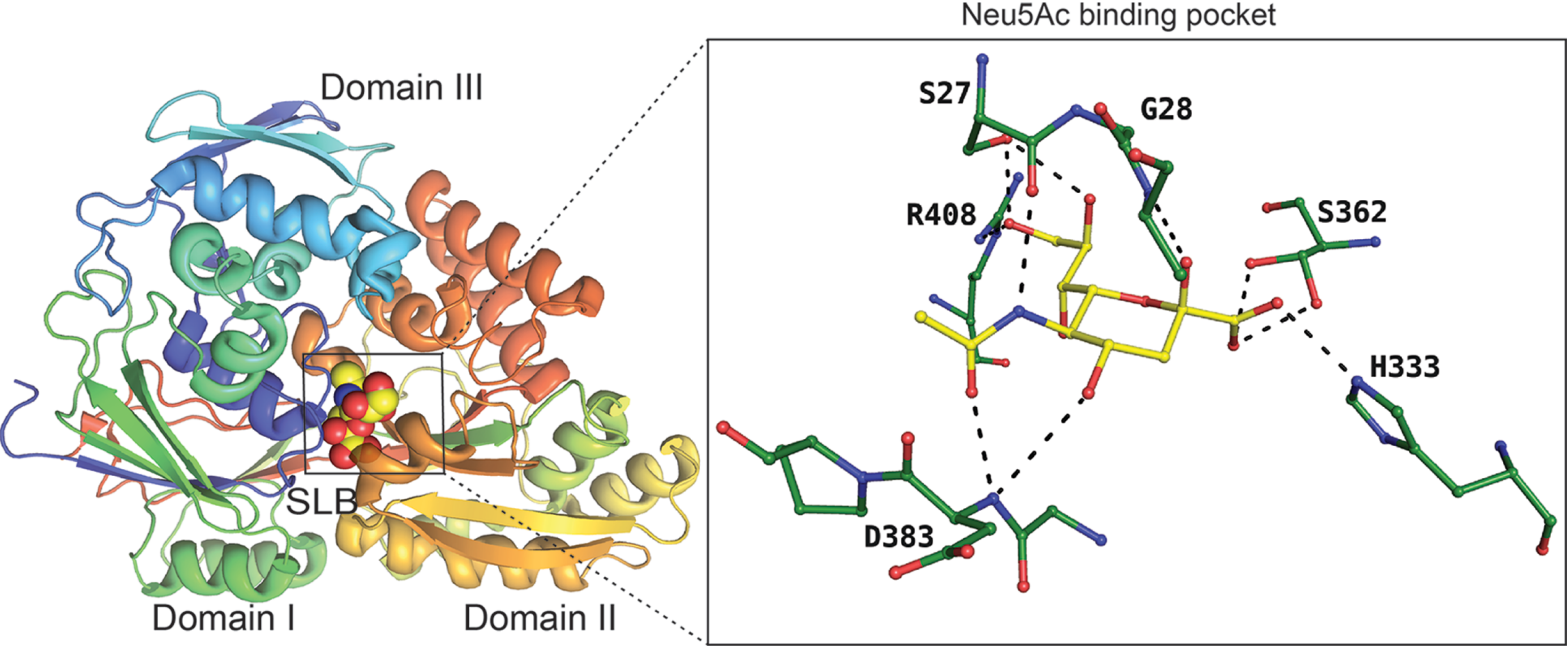
SatA binding to the β-anomer of Neu5Ac (SLB), represented by spheres. The insert displays the binding pocket of SatA and the residues interacting with Neu5Ac (PDB entry 5z99; Gangi Setty *et al.*, 2018 ▸), represented as a ball-and-stick diagram.

*Haemophilus ducreyi* is a Gram-negative coccobacillus that belongs to the *Pasteurellaceae* family and causes sexually transmitted chancroid disease. It has been shown that the LOS of *H. ducreyi* also acts as a putative virulence factor and is heavily sialylated. It has been shown that *H. ducreyi* uses a novel ABC-transport system to transport sialic acid encoded by the gene SatABCD (Post *et al.*, 2005 ▸). SatA encodes a periplasmic binding protein that binds sialic acid, and SatBCD encodes an integral membrane protein with a nucleotide-binding domain in this operon. No structure off the ABC-type Neu5Ac transporter (SatBCD) has been reported. The periplasmic binding protein SatA has been well studied (Setty *et al.*, 2018 ▸). Substrate-bound *Hd*-SatA acts as an anchor for the SatBCD membrane transporter, facilitating the transport of Neu5Ac/Neu5Gc. *Hd*-SatA is an α/β protein comprising three domains, with the N- and C-terminal domains connected by two β-strands serving as a hinge region. Interestingly, multiple main-chain atoms interact with the polar groups of Neu5Ac. The only residue that could neutralize the charge on the carboxyl­ate group is histidine 333 (Fig. 3 ▸ insert). On ligand binding, domains I and II converge to trap the ligand, which is reminiscent of the Venus flytrap mechanism (Setty *et al.*, 2018 ▸).

### 3.2. Secondary active transporters

Secondary active transport is a mechanism in which solutes are transported across a membrane owing to the difference in the electrochemical gradient established by the transport of ions such as H^+^ or Na^+^ across the membrane. The three main families in this category are the major facilitator superfamily (MFS), the SSS and TRAP transporters.

#### 3.2.1. SSS transporter

**Figure 4:**
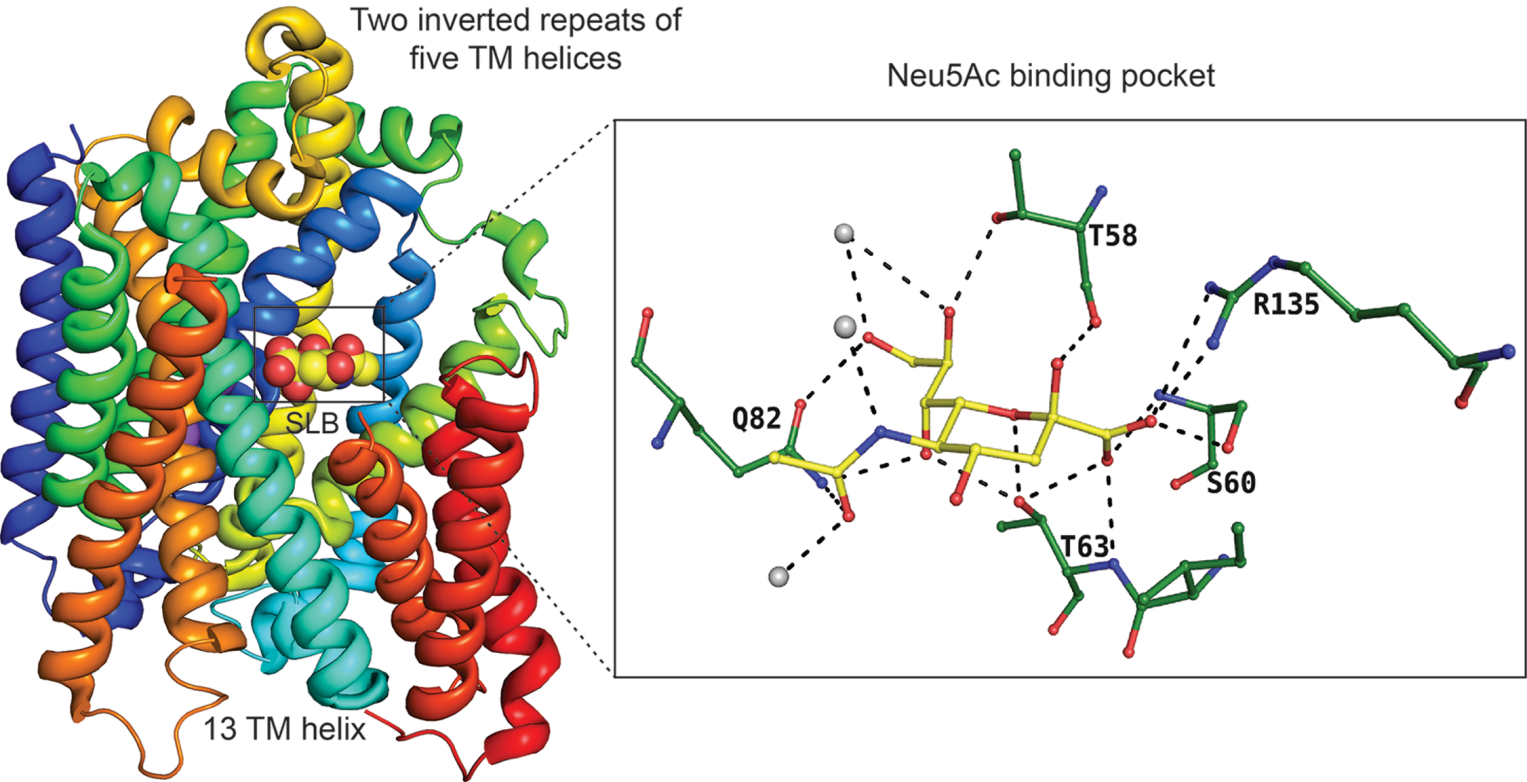
SSS transporters binding to the β-anomer of Neu5Ac, represented by spheres. The ball-and-stick diagram displays residues interacting with Neu5Ac (PDB entry 5nva; Wahlgren *et al.*, 2018 ▸). Solvent molecules are shown as grey spheres.

In the SSS transport system, an Na^+^ ion electrochemical gradient is used to uptake sialic acids. The Na^+^/galactose/glucose symporter from *Vibrio parahaemolyticus* (vSGLT) was the first representative of the SSS transport family (Faham *et al.*, 2008 ▸). Uropathogenic *Proteus mirabilis* transports and catabolizes host-derived sialic acids using the SSS transport system (SiaT). Functional and molecular dynamics studies of SiaT have shown that the uptake and transport of sialic acid are also controlled by two Na^+^ (Na2 and Na3) ions (Wahlgren *et al.*, 2018 ▸). The high-resolution structure of the outward-facing and substrate-bound form of the SiaT sialic acid transporter from *Proteus mirabilis* (*Pm*–SiaT), a member of the SSS family, has been elucidated previously (Wahlgren *et al.*, 2018 ▸). *Pm*–SiaT adopts the LeuT-fold, with Neu5Ac bound centrally and two Na^+^ ions facilitating its transport. Comprising 13 transmembrane helices, *Pm*–SiaT positions its N- and C-termini towards the periplasmic and cytoplasmic regions, respectively (Fig. 4 ▸). Two inverted repeats of five transmembrane helices form a core structural fold. Structural studies show that Na2 occupies a conserved site and Na3 occupies a unique position, which is present at ∼14 Å from the substrate-binding site and 6.5 Å away from Na2. Microscale thermophoresis experiments revealed that *Staphylococcus aureus* SiaT exhibits a significantly higher affinity for Neu5Gc sialic acid (*K*_d_ = 39 ± 5 µ*M*) than for Neu5Ac (*K*_d_ = 113 ± 6 µ*M*) (North, Wahlgren *et al.*, 2018 ▸). Interestingly, *Pm*–SiaT displays a reversed preference, exhibiting a slightly higher binding affinity for Neu5Ac (*K*_d_ = 58 ± 1 µ*M*) than for Neu5Gc (*K*_d_ = 85 ± 2 µ*M*). Neu5Ac is stabilized at its binding pocket by a number of polar interactions. There are no water-mediated polar interactions on the C1 side of the Neu5Ac, suggesting strong electrostatic interactions. The C9 and C5 extensions have a number of water-mediated interactions that decrease the contribution of electrostatics towards binding affinity in these regions.

#### 3.2.2. MFS transporters

MFS is one of the major transport systems found in bacteria, archaea and eukaryotes. Unlike ABC transporters, which transport both small molecules and macromolecules, MFS transporters only transport small solutes in response to the electrochemical ion gradient. Based on the direction of solute/ion transport, MFS transporters are classified as uniporters, symporters or antiporters. They transport essential nutrients and export toxic substances or end products of metabolism. NanT, which transports sialic acid, is part of the MFS transport system. NanT was first identified in *E. coli* and its mutation leads to a loss of function. Unlike traditional MFS family members, this protein contains 14 predicted transmembrane helices and an amphiphilic α-helix that might play an important role in the structure and function of the protein (Martinez *et al.*, 1995 ▸).

#### 3.2.3. TRAP transporters

**Figure 5:**
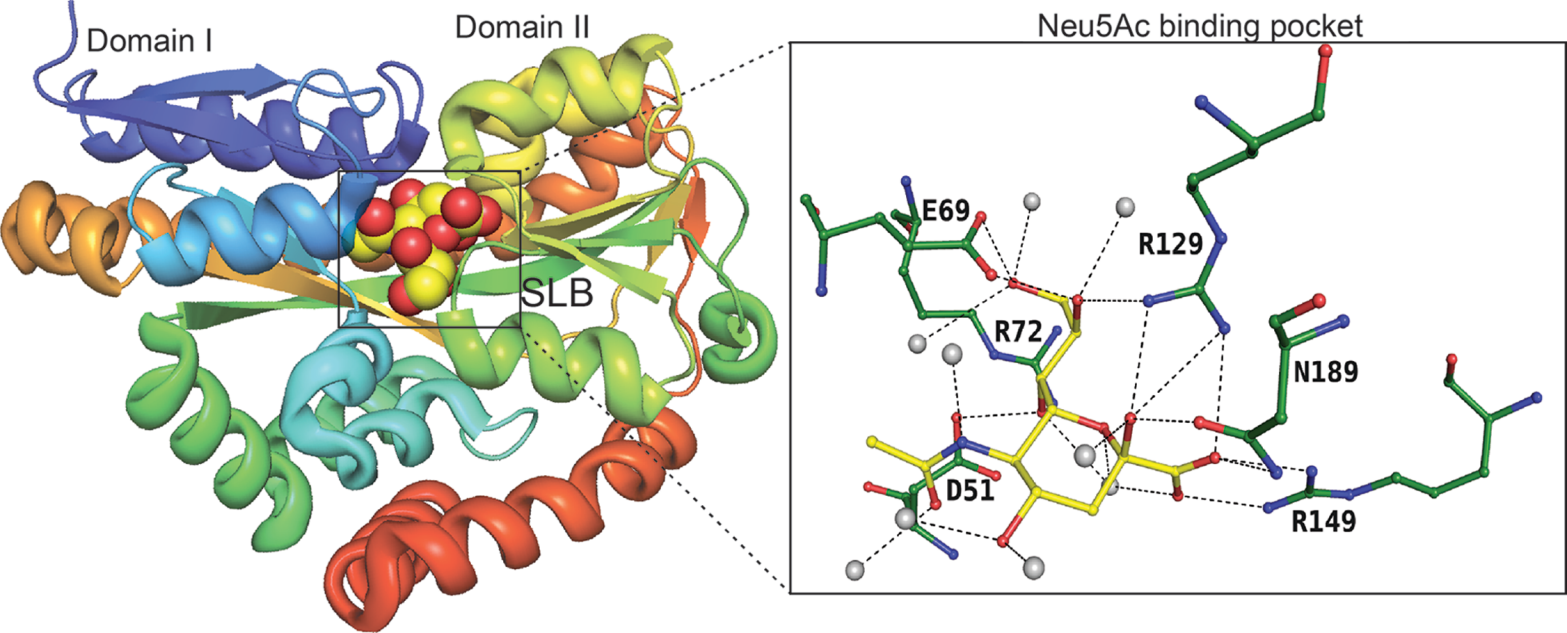
SiaP binding to the β-anomer of Neu5Ac, represented by spheres. As a ball-and-stick diagram, the insert shows the binding site with the residues involved in the interaction with Neu5Ac (PDB entry 4mmp; Gangi Setty *et al.*, 2014 ▸). Solvent atoms are represented as grey spheres.

TRAP transporters are extracytoplasmic solute receptors (ESRs) that belong to the category of secondary transporters and are present only in bacteria and archaea. Similar to the MFS transport system, the electrochemical ion gradient provides a driving force for solute transport across the membrane. Unlike the conventional MFS and SSS transport systems, the TRAP transport system contains an extra substrate binding protein (SBP) to trap the substrate in the periplasm and confers unidirectionality to substrate transport (Mulligan *et al.*, 2009 ▸). The TRAP transport system is a three-component system consisting of an extracellular solute receptor (SiaP, periplasmic binding protein) and two unequal-sized distinct integral membrane proteins (Q and M domains). The periplasmic binding protein SiaP delivers Neu5Ac to the TRAP transporter and binds to Neu5Ac with nanomolar affinity. The sequence similarity between SatA and other SiaPs that bind to sialic acids is approximately 20–25%. In Gram-negative bacteria, solute-binding proteins are present in the periplasm, whereas in Gram-positive bacteria, they are anchored to the inner membrane of the bacteria. Several periplasmic sialic acid-binding proteins have been shown to bind to Neu5Ac (North, Horne *et al.*, 2018 ▸; Gangi Setty *et al.*, 2014 ▸). The SiaP protein of *H. influenzae*, referred to as *Hi*–SiaP, is a prominent example of a sialic acid-binding protein within the TRAP transport system. Initially, Müller *et al.* (2006 ▸) elucidated the structure of *Hi*–SiaP bound to the sialic acid analog 2,3-dide­hydro-2-de­oxy-*N*-acetyl­neuraminic acid (Neu5Ac2en), revealing a two-domain protein configuration, followed by the demonstration that Neu5Ac binds enthalpically and with nanomolar affinity to SiaPs (Fig. 5 ▸) (Johnston *et al.*, 2008 ▸). The enthalpic component of binding is not surprising given the large number of electrostatic charge interactions in the binding pocket. Mutations of R149 and R129 have been shown to decrease the binding affinity (Gangi Setty *et al.*, 2014 ▸).

Recently, membrane protein components of two TRAP transporter protein structures have been reported, one from *Haemophilus influenzae* and the other from *Photobacterium profundum*, using cryo-EM (Davies *et al.*, 2023 ▸; Peter *et al.*, 2022 ▸; Currie *et al.*, 2023 ▸). The structure revealed that the protein was in an inward-open conformation with two sodium sites. We recently determined the TRAP structure from *Fusobacterium nucleatum* at 2.8 Å resolution. The *Fn*–SiaQM structure was in an inward-open conformation with three sodium sites.

## 4. Sialic acid catabolism pathway

**Figure 6:**
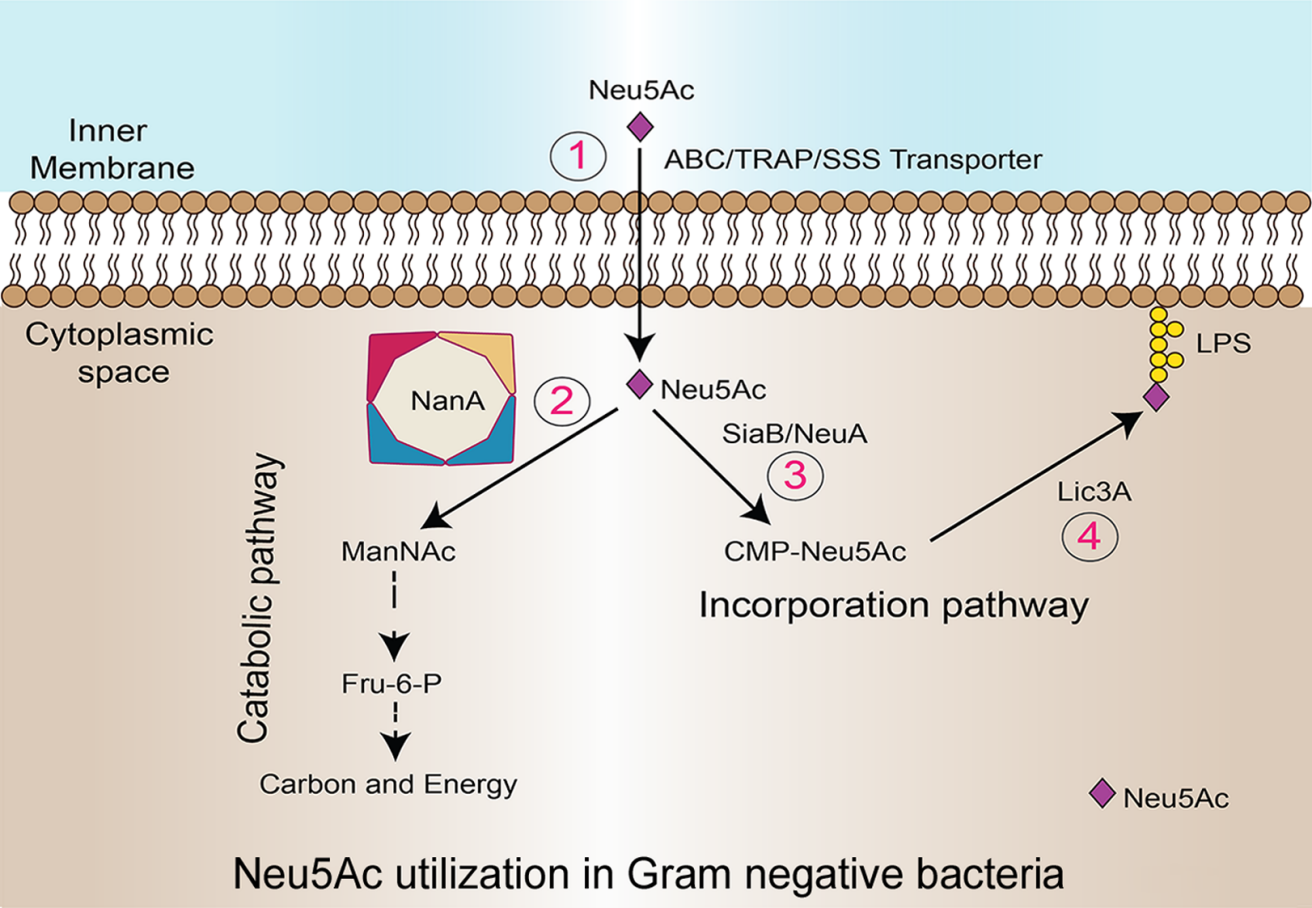
Illustration of the Neu5Ac utilization pathway. (1) Various transporter proteins facilitate the movement of Neu5Ac from the periplasmic space into the cytoplasm. (2) In the catabolic pathway, the first enzyme, NanA (Neu5Ac lyase), initiates the utilization of Neu5Ac. (3) SiaB (CMP–Neu5Ac synthetase) adds CMP to Neu5Ac in the incorporation pathway. (4) Lic3A (sialyltransferase) incorporates Neu5Ac into the LPS.

Once β-Neu5Ac is present in the cytoplasm, it can be used as a carbon or nitro­gen source (Fig. 6 ▸). The first evidence that bacteria utilize sialic acids as the sole carbon source came from studies on *Clostridium perfringens* (Nees *et al.*, 1976 ▸). Further studies have shown that only a limited number of commensals and pathogenic bacteria that are closely associated with the host have genes for sialic acid catabolism. *H. influenzae*, *F. nucleatum*, *P. multocida*, *S. aureus*, *V. cholera*, *V. vulnificus* and *E. coli* have been shown to possess a nan cluster of genes (Olson *et al.*, 2013 ▸; Almagro-Moreno & Boyd, 2009 ▸). Alternatively, cytidine-5′-monophosphate (CMP) is utilized as a substrate by sialic acid synthetase enzymes, and catalyzes the conversion of CMP and siliac acid to cytidine-5′-monophos­phate–*N*-acetyl­neuraminic acid (CMP–Neu5Ac), which is a key intermediate in the sialic acid biosynthetic pathway. Neu5Ac (from CMP-Neu5Ac) is incorporated into lipopolysaccharide (LPS) molecules through the action of sialyltransferase enzymes. Sialyltransferases are glycosyl­transferases that catalyze the transfer of sialic acid residues from CMP–Neu5Ac to terminal sugar residues on glycoconjugates such as LPS. During this process, the sialyltransferase enzyme binds to both the CMP–Neu5Ac substrate and the acceptor molecule, which typically contains a terminal galactose or *N*-acetyl­galactosamine residue (Mizanur & Pohl, 2008 ▸). The enzyme then facilitates the transfer of the sialic acid residue from CMP–Neu5Ac to the terminal sugar residue on the acceptor molecule, forming a glycosidic bond and thereby incorporating the sialic acid moiety into the LPS molecule. LPS sialylation can have significant effects on bacterial physiology, including host–pathogen interactions, immune evasion and bacterial virulence.

### 4.1. Regulation of sialic acid catabolic genes

**Figure 7:**
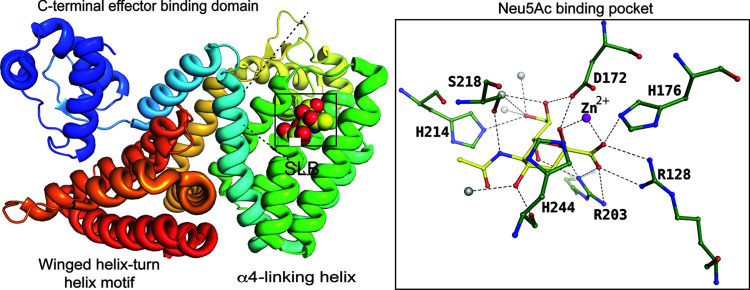
NanR binding to the β-anomer of Neu5Ac, represented by spheres. The insert displays the binding site of NanR, showing the residues involved in the interaction with Neu5Ac (PDB entry 6on4; Kalivoda *et al.*, 2013 ▸) as a ball-and-stick diagram. Solvent atoms are represented as grey spheres and the zinc atom as a purple sphere.

NanR is a transcriptional regulator (repressor) involved in the regulation of genes related to sialic acid metabolism in bacteria such as *E. coli*. The mechanism underlying NanR gene repression and allosteric induction of bacterial sialic acid metabolism involves intricate molecular interactions between NanR, DNA operator sites and sialic acid molecules, such as Neu5Ac (Fig. 7 ▸). The dissociation constant (*K*_D_) for Neu5Ac binding to NanR determined using isothermal titration calorimetry yielded a consistent *K*_d_ of 16 µ*M*, with one Neu5Ac molecule binding per NanR dimer (Horne *et al.*, 2021 ▸). The presence of Zn^2+^ in the binding pocket influences the impact of the charged residues. There is a salt bridge formed between C1-carboxyl­ate and R128, and another electrostatic interaction with R203. However, the *K*_d_ value does not reflect nanomolar affinity, as Zn^2+^ can affect the charge on the C1-carboxyl­ate.

### 4.2. Conversion of Neu5Ac into fructose-6-phosphate

**Figure 8:**
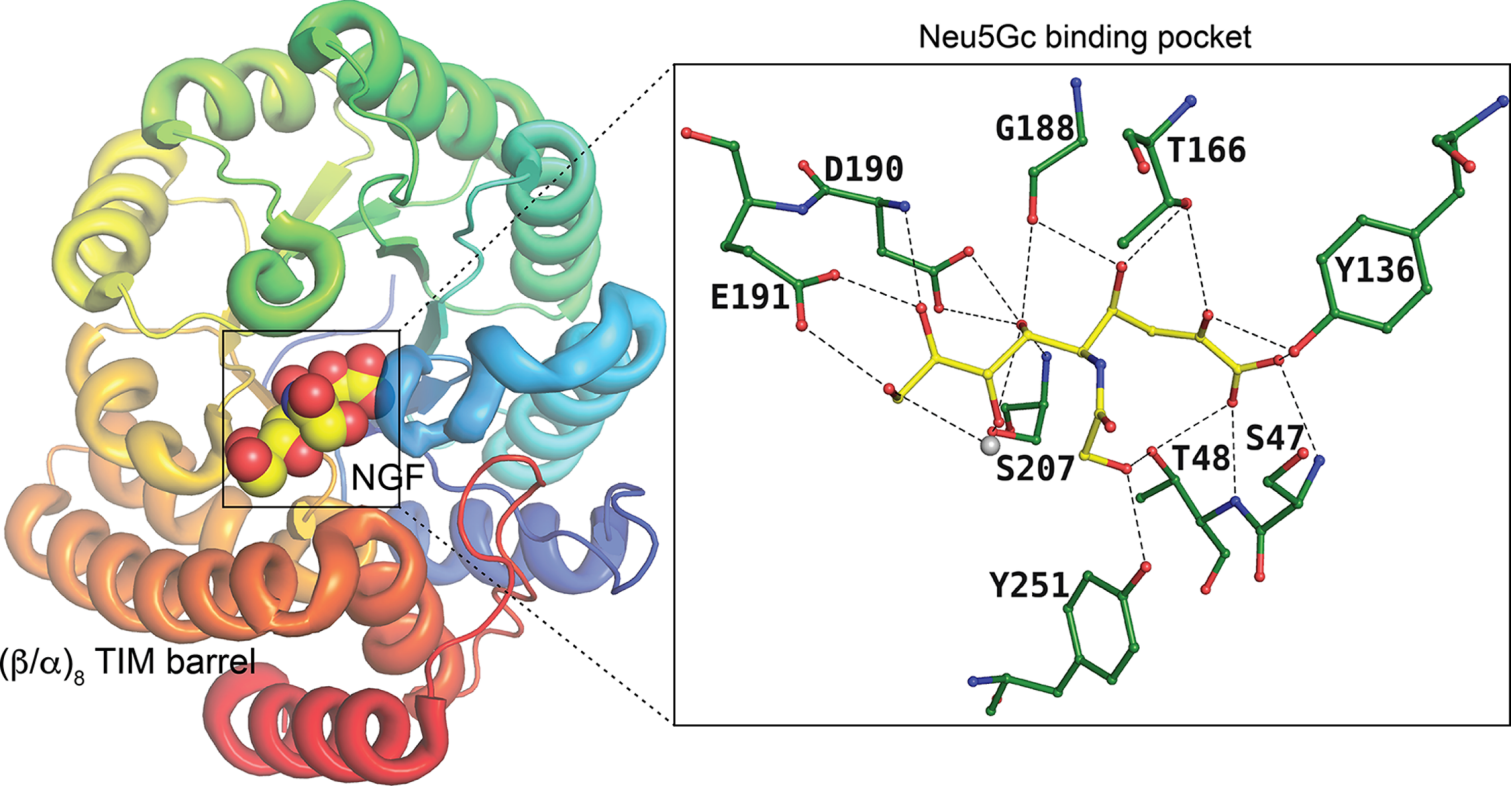
NanA binding to the Neu5Gc, represented as spheres. The insert displays the active site of NanA and the residues involved in the interaction with Neu5Gc (PDB entry 4img; Huynh *et al.*, 2013 ▸) as a ball-and-stick diagram. Note that the sugar is the linear form needed for the lysis of the pyruvate moiety. The solvent atom is shown in grey.

Five enzymes govern catabolism, with *N*-acetyl­neuraminate lyase (NanA) being the first committed enzyme. NanA cleaves Neu5Ac into *N*-acetyl-d-mannosamine (ManNAc) and pyruvate, initiating this pathway. Enzymes including *N*-acetyl­mannosamine kinase (NanK), *N*-acetyl­mannosamine-6-phos­phate epimerase (NanE), *N*-acetyl­glucosamine-6-phosphate de­acetyl­ase (NagA) and glucosamine-6-phosphate deaminase (NagB) further metabolize the resulting products. NanA, also known as sialic acid aldolase, is present in both pathogenic and non-pathogenic bacteria and in various mammalian tissues and plays a vital role in the metabolism of sialic acids. NanA, which belongs to the class I aldolase family, exhibits a triosephosphate isomerase (TIM)-barrel fold and employs aldol condensation by forming a Schiff base intermediate. The high-resolution structures of *N*-acetyl­neuraminate lyase from *F. nucleatum*, *H. influenzae*, *E. coli*, *S. aureus* and *P. multicoda* revealed that NanA adopts a classical TIM (triosephosphate isomerase) barrel tertiary structure fold (Campeotto *et al.*, 2009 ▸; North *et al.*, 2016 ▸; Kumar *et al.*, 2018 ▸) with the addition of three extra helices (I, J and K) at the C-terminus (Fig. 8 ▸). Sialic acid binds in the extended conformation (against the pyran­ose form in the other cases), and there are no basic residues that are close to the C1-carboxyl­ate group. The structures and functions of the other enzymes have also been well characterized. As they do not bind to sialic acid, their structures and properties are not discussed in this review.

### 4.3. Incorporation of Neu5Ac as the outermost sugar of LPS

**Figure 9:**
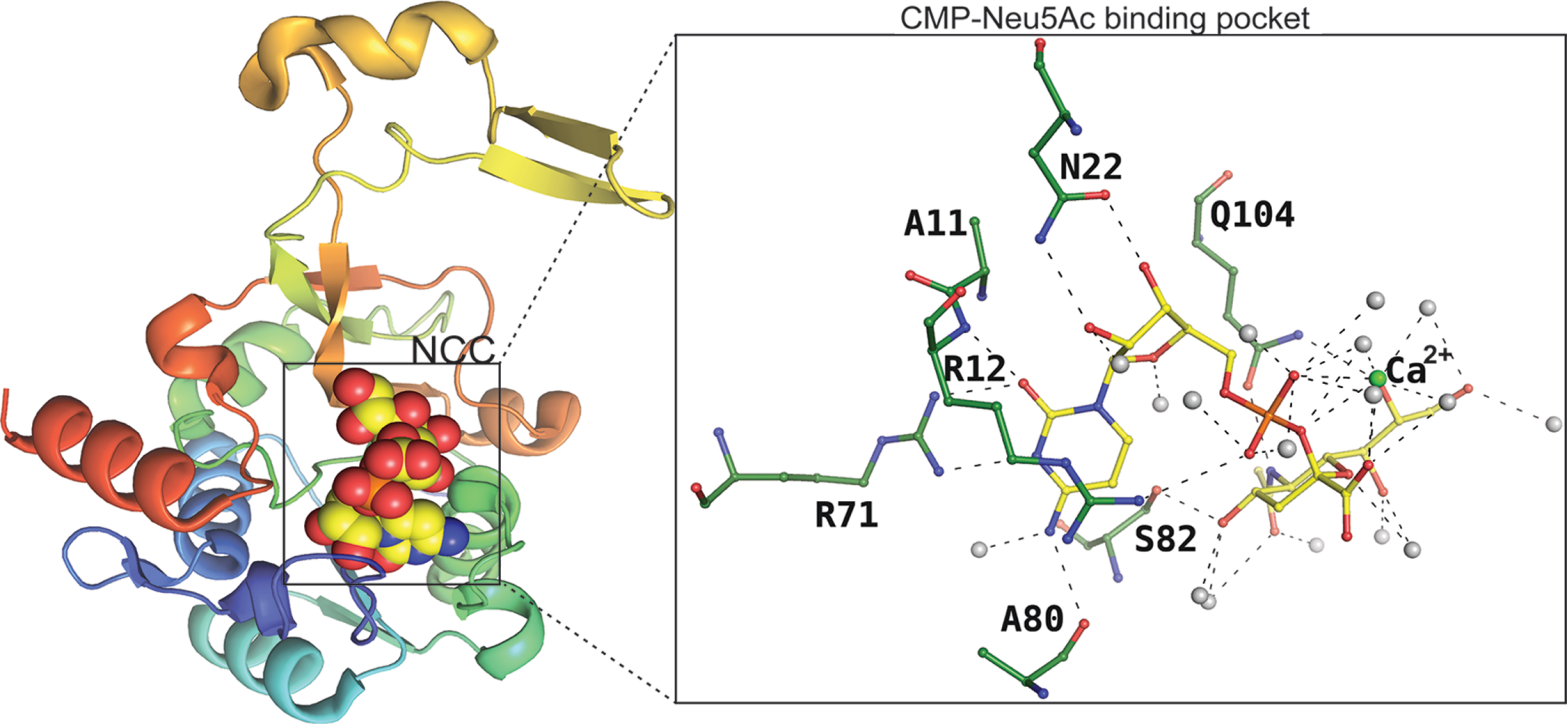
SiaB binding to the CMP–Neu5Ac, represented as spheres. The insert displays the active site of SiaB, highlighting the residues involved in the interaction with CMP–Neu5Ac (PDB entry 6ckm; Matthews *et al.*, 2020 ▸) as a ball-and-stick diagram. Solvent atoms are represented as grey spheres and the calcium atom is shown as a green sphere.

Most pathogenic bacteria incorporate Neu5Ac onto their surfaces to evade the host immune system. The primary enzymes responsible for constructing and synthesizing Neu5Ac-containing glycoconjugates are sialyltransferases, which utilize CMP–Neu5Ac as an activated sugar nucleotide donor substrate (Bose *et al.*, 2019 ▸). CMP-sialic acid synthetases (CSSs) facilitate the activation of the β-anomer of free Neu5Ac, forming a β-linked sialyl monophosphate diester bond between the C2 of Neu5Ac and the α-phosphate of cytidine-5′-triphosphate (CTP), subsequently releasing pyrophosphate (Fig. 9 ▸). Fig. 9 ▸ also suggests that the specificity of binding probably originates from the nucleotide as there are more direct interactions with the side chain of the protein. Neu5Ac interactions occur through the solvent molecules. The next enzyme binds to CMP–Neu5Ac and transfers Neu5Ac to the outermost sugar of LPS.

## 5. Discussion and conclusions

As we suggested in the introduction[Sec sec1], we hoped that of the two periplasmic binding proteins that sequester Neu5Ac, the three types of transport proteins, the transcription factor that binds to sialic acid and the two enzymes that bind to Neu5Ac, a few would have a common binding motif. This would allow us to pursue a drug discovery program targeting multiple proteins in the same pathogen, thereby reducing the ability to acquire resistant mutations. However, as described above, these proteins bind differently to Neu5Ac.

**Figure 10:**
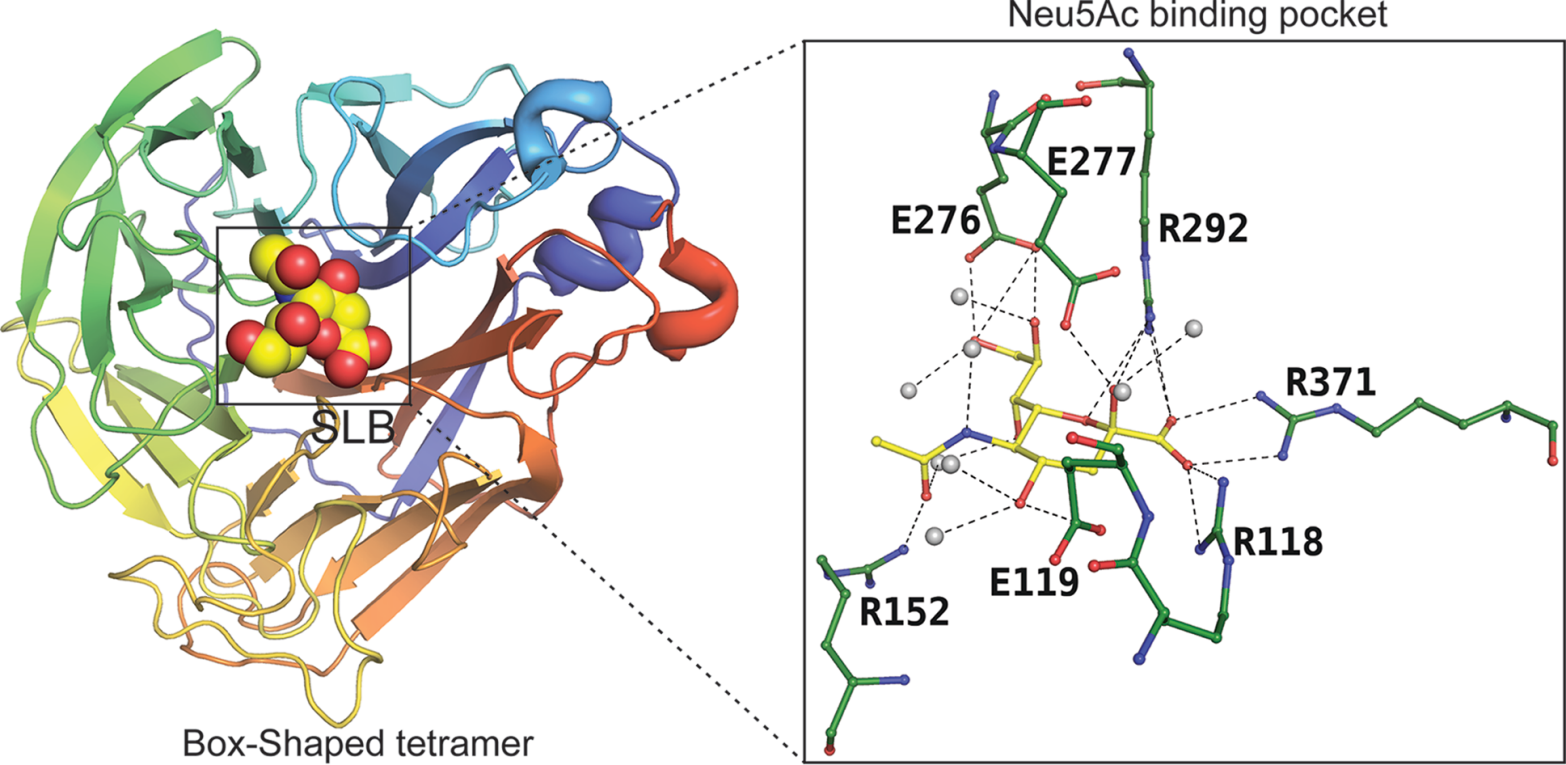
Neuraminidase binding to the β-anomer of Neu5Ac, represented by spheres. The insert displays the active site of neuraminidase, highlighting the residues involved in the interaction with Neu5Ac (PDB entry 4h53; Vavricka *et al.*, 2013 ▸) as a ball-and-stick diagram. Solvent atoms are represented as grey spheres.

We searched for other proteins in the Protein Data Bank (PDB) with bound β-Neu5Ac and classified them based on their structural homology and binding pocket topology. Here, we discuss examples of unique binding pockets. Two major surface antigenic proteins are present in influenza viruses: hemagglutinin (HA) and sialidase (neuraminidase). Their roles in the influenza virus infection cycle are well understood. Neuraminidases of viral origin have been well studied, and inhibitors of influenza virus neuraminidase are currently available as antiviral agents (Gubareva & Mohan, 2022 ▸). The structure and mode of binding of neuraminidase (Fig. 10 ▸) show a number of polar interactions (Vavricka *et al.*, 2013 ▸), with some elements common to that of the SiaP of the TRAP transport system.

**Figure 11:**
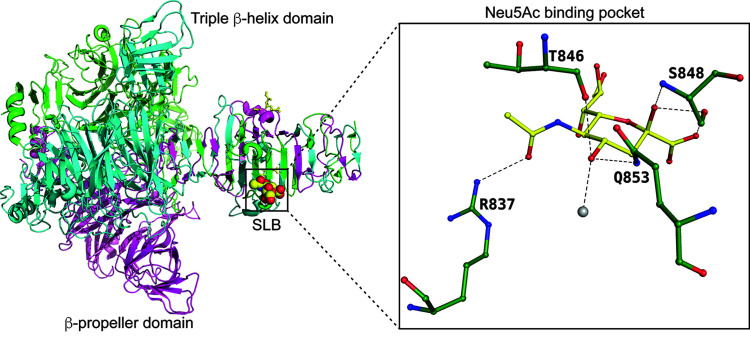
Binding of bacteriophage endosialidase to Neu5Ac, represented as spheres. The insert displays the active site of endosialidase, highlighting the residues involved in the interaction with Neu5Ac (PDB entry 1v0f; Stummeyer *et al.*, 2005 ▸) as a ball-and-stick diagram. The solvent atom is represented as a grey sphere.

Pathogenic bacteria use molecular mimicry to infiltrate and establish themselves within their hosts, often by mimicking host structures, notably those present in capsular polysaccharides. *E. coli* K1, a neuroinvasive bacterium responsible for neonatal sepsis and meningitis, employs this strategy prominently. Its capsule comprises poly α2,8-sialic acid (polySia). This sugar polymer acts as a critical modulator of neuronal plasticity in adult human hosts and evades the mammalian immune system, making the host tolerant to polySia-shielded bacteria. Several coliphages that infect polySia-encapsulated *E. coli* K1 have been identified, each harbouring endosialidase tail spikes which are crucial for infection. Cloned from various K1-specific phages, these *endo*-sialidases belong to the GH-58 family of glycosyl­hydro­lases and exhibit a modular composition with a highly conserved catalytically active central region and distinctive N- and C-terminal domains. The tertiary structures of *endo*-sialidases from phages differ greatly (Stummeyer *et al.*, 2005 ▸) (Fig. 11 ▸). However, they interact with fewer polar interactions, and the orientation of the different interacting residues is different from that of the neuraminidases.

**Figure 12:**
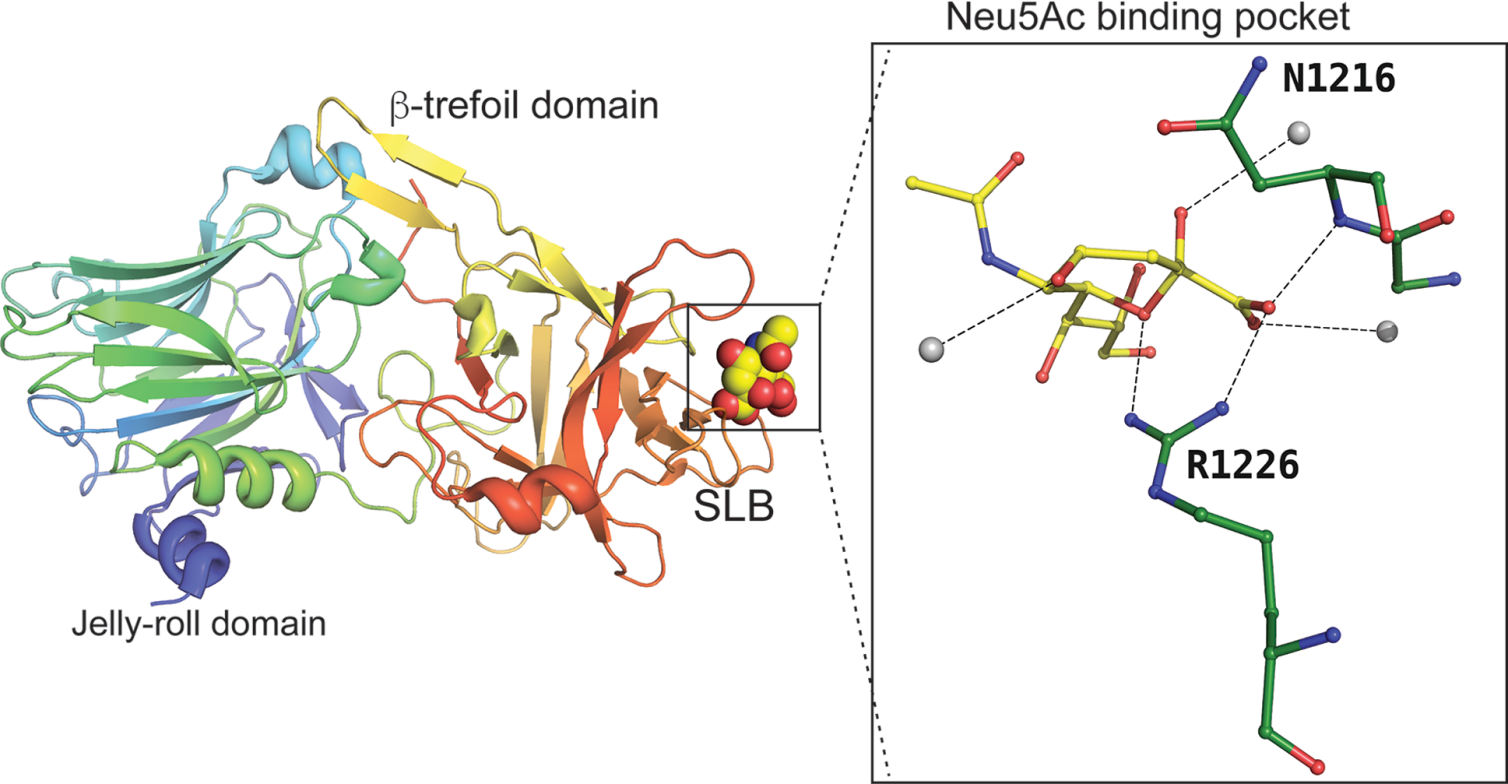
Botulinum neurotoxin binding to Neu5Ac. Insert: binding site of botulinum neurotoxin and residues interacting with Neu5Ac (PDB entry 1dfq; Emsley *et al.*, 2000). Solvent molecules are represented as grey spheres.

Botulinum neurotoxins (BoNTs) produced by *Clostridium botulinum*, are potent inhibitors of synaptic transmission in the peripheral cholinergic nervous system (Montal, 2010 ▸). Neurotoxins bind to Neu5Ac, and their binding sites have been characterized (Fig. 12 ▸). Here, Neu5Ac was bound to the surface (Emsley *et al.*, 2000 ▸). Arg 1226 is the residue anchoring the orientation, with direct hydrogen bonds to the carboxyl­ate atom and the pyran­ose oxygen.

**Figure 13:**
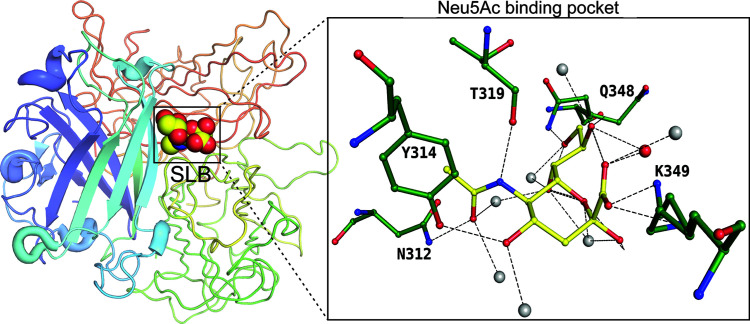
Adenovirus fibre knob protein binding to Neu5Ac. Insert: binding site of the adenovirus fibre knob protein and residues interacting with Neu5Ac (PDB entry 6qu8; Baker *et al.*, 2019 ▸). Solvent atoms are represented as grey spheres.

The last example is that of the adenovirus fibre knob protein that binds to Neu5Ac as well (Baker *et al.*, 2021 ▸). Here, the protein forms a trimer (a cartoon for only one subunit is shown), and three molecules of Neu5Ac bind in between two subunits (Fig. 13 ▸). As the binding occurs at the interface, Neu5Ac binds to residues on two different subunits. The authors modelled the Lys349 in multiple conformations, even though the terminal amino group hydrogen bonds to the C1-carboxyl­ate oxygen in both conformations.

**Figure 14:**
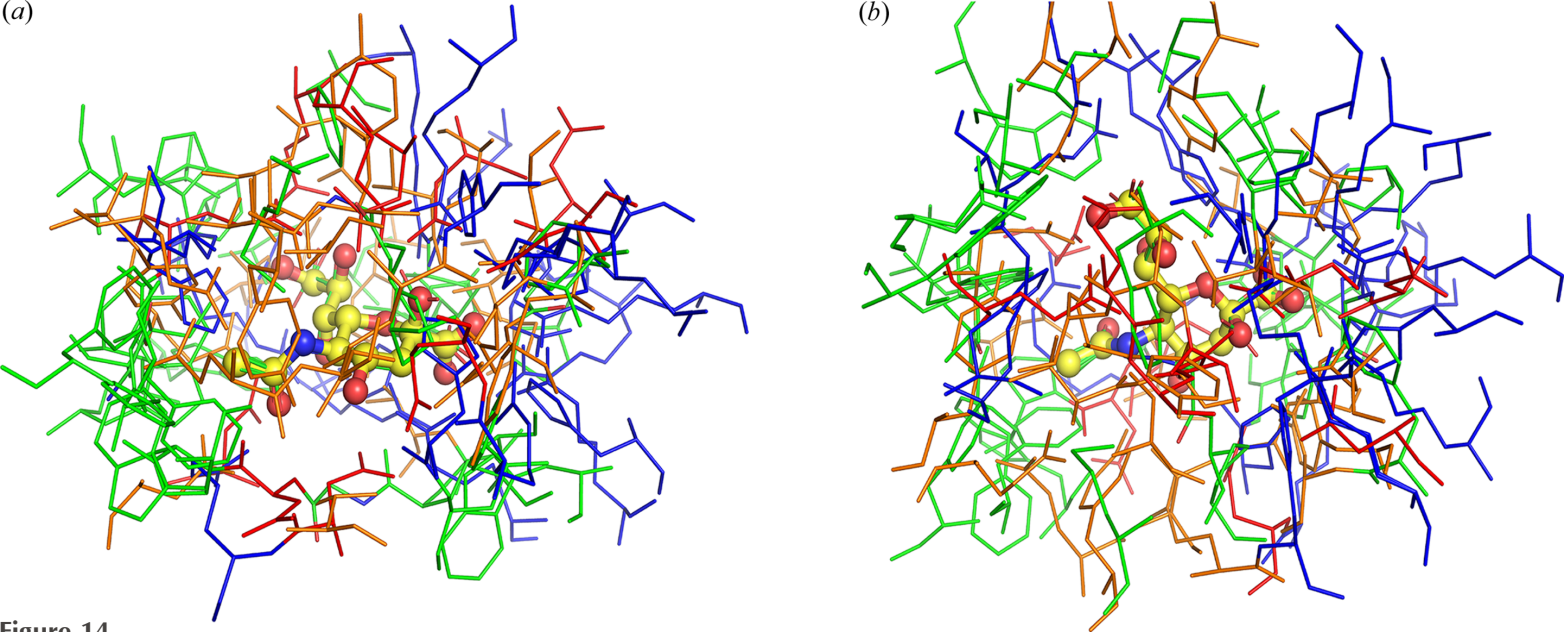
Superposition of Neu5Ac in different structures described in this review, where β-Neu5Ac exists in the pyran­ose form: (*a*) and (*b*) are two views rotated 90°. The C1-carboxyl­ate is on the right. Arg, Lys and His are represented as blue lines; Asp and Glu are red; polar residues Ser, Thr, Asn, Gln and Tyr are orange; and the non-polar residues Met, Phe, Pro, Trp, Val, Leu, Ile and Ala are green.

Figs. 3 ▸–5 ▸ ▸ and 7 ▸–13 ▸ ▸ ▸ ▸ ▸ ▸ illustrate the different modes of β-Neu5Ac binding to different proteins. The different tertiary structures of the binding motifs clearly show that they evolved independently. This hypothesis was also based on sequence comparisons (Severi *et al.*, 2021 ▸). However, one could hypothesize convergent evolution of different proteins to organize similarities in the binding motif of Neu5Ac. However, this is not the case. If we organize all the PDB files of the ten different motifs that have been reported and superpose Neu5Ac, it is clear that some interactions occur more often than others (Fig. 14 ▸). The C1-carboxyl­ate, in several cases, interacts with positively charged Arg or Lys. This is not surprising; however, it is surprising that in several cases, there seems to be no obvious charge neutralization. We used all of the structures except lyase and the CMP–sialic acid synthase. *PyMOL* was used to superimpose sialic acid coordinates (version 1.8; Schrödinger LLC, 2015). The figure shows that Neu5Ac superposes well and that there are minor rotations around the free single bonds. Neu5Ac is shown as a stick for only one structure (Fig. 14 ▸). The surrounding residues are shown in lines. There are over 40 structures in the PDB that contain bound β-Neu5Ac. We used only one representative of each binding mode to illustrate that the different binding modes are distinct. All superposed structures were in the pyran­ose form. The enzyme NanA, which is a lyase, binds to sugar in the open-chain form (Fig. 8 ▸) and is not included in the figure and analysis. Although the figure looks like all the residues are randomly oriented, there is a cluster of basic residues around the C1-carboxyl­ate (blue lines on the right side of the figure). Although there are exceptions, this may be necessary to neutralize the negative charge at the physio­logical pH of the carboxyl­ate. Similarly, a less populated cluster of hydro­phobic residues is on the opposite side (around the *N*-acetyl part of the sugar).

Bhagavat & Chandra (2014 ▸) published an analysis of Neu5Ac binding sites and classified them into six groups. Numerous additional structures have been reported in subsequent studies. In this review, we have demonstrated the existence of at least ten distinct classes of binding sites. In summary, we see the evolutionary origins of various proteins that engage with Neu5Ac lead to diverse mechanisms through which nature achieves specific binding to this ligand. Although the lock-and-key concept is fascinating for ligand binding, nature has evolved multiple locks for a single key. Attempts to synthesize tight binding inhibitors towards one target that binds to sialic acid do not automatically result in binding to other sialic acid-binding proteins (Bozzola *et al.*, 2022 ▸). This prompts questions about the insights that can be gained for designing a molecule capable of targeting multiple active sites simultaneously.
